# The challenge of segmental small bowel motility quantitation using MR enterography

**DOI:** 10.1259/bjr.20140330

**Published:** 2014-07-14

**Authors:** A Menys, A Plumb, D Atkinson, S A Taylor

**Affiliations:** Centre for Medical Imaging, University College London, UK.

## Abstract

**Objective:**

Analysis of “cine” MRI using segmental regions of interest (ROIs) has become increasingly popular for investigating bowel motility; however, variation in motility in healthy subjects both within and between scans remains poorly described.

**Methods:**

20 healthy individuals (mean age, 28 years; 14, males) underwent MR enterography to acquire dynamic motility scans in both breath hold (BH) and free breathing (FB) on 2 occasions. Motility data were quantitatively assessed by placing four ROIs per subject in different small bowel segments and applying two measures: (1) contractions per minute (CPM) and (2) Jacobian standard deviation (SD) motility score. Within-scan (between segment) variation was assessed using intraclass correlation (ICC), and repeatability was assessed using Bland–Altman limits of agreement (BA LoA).

**Results:**

*Within-scan segmental variation*: BH CPM and Jacobian SD metrics between the four segments demonstrated ICC *R* = 0.06, *p* = 0.100 and *R* = 0.20, *p* = 0.027 and in FB, the CPM and Jacobian SD metrics demonstrated ICC *R* = −0.26, *p* = 0.050 and *R* = 0.19, *p* = 0.030. *Repeatability*: BH CPM for matched segments ranged between 0 and 14 contractions with BA LoA of ±8.36 and Jacobian SD ranged between 0.09 and 0.51 with LoA of ±0.33. In FB data, CPM ranged between 0 and 10 contractions with BA LoA of ±7.25 and Jacobian SD ranged between 0.16 and 0.63 with LoA = ±0.28.

**Conclusion:**

The MRI-quantified small bowel motility in normal subjects demonstrates wide intersegmental variation and relatively poor repeatability over time.

**Advances in knowledge:**

This article presents baseline values for healthy individuals of within- and between-scan motility that are essential for understanding how this process changes in disease.

Dynamic “cine” MRI acquired during MR enterography is increasingly utilized to assess bowel motility in a range of conditions, notably inflammatory bowel disease and enteric dysmotility syndromes.^1–4^ Analysis of the data remains primarily subjective in clinical routine, but the ability to apply quantitative techniques makes this a potentially powerful methodology to explore gastrointestinal physiology in disease as well as an emerging application as a biomarker for drug efficacy.^5–7^

Despite the growing literature, a consensus has yet to be reached as to the best method of quantitatively analysing small bowel data and indeed a range of motility metrics are proposed.^2,3,8–12^ The most commonly used metric is the change in luminal diameter at a fixed anatomical position through the time series. By tracking bowel diameter, a characteristic curve can be produced with the number of contractions expressed per minute (CPM) to give an intuitive and broadly accepted metric for small bowel motility (SBM).^2–4,9,11,13–15^ To date, several studies have reported a relationship between CPM and dysmotility in disease, either compared with a histopathological standard or “normal” reference bowel loops.^2–4,12^ An array of additional metrics derived both from bowel diameter measures and more abstract processing techniques have further been implemented with varying degrees of effectiveness in disease and health.^2,4,5,8,10,14,16^

Although intuitively attractive, the robustness of assessing overall enteric motility using only an isolated loop of bowel has received relatively little attention to date irrespective of the precise metric applied. It is unclear how representative the selected bowel loops are of overall SBM and if normal motility intrinsically differs between bowel segments, for example, between the jejunum and ileum. Furthermore, the repeatability of single loop metrics, even in normal individuals, is not well described, knowledge of which is vital if segmental analysis is to be used to diagnose, guide treatment and monitor enteric pathology.

The purpose of this study is to explore segmental variation in SBM in healthy volunteers measured using two commonly reported small bowel metrics [CPM and Jacobian standard deviation (SD)] looking at (1) within-scan motility variation between different segments and (2) between-scan variation (repeatability) across two time points.

## METHODS AND MATERIALS

Full ethical permission was obtained for the study from the relevant research ethics committee. All subjects provided written consent.

### Patients

20 healthy volunteers (mean age, 28 years; range, 22–48 years; 14 males) were recruited over an 18-month period. Each subject underwent MRI enterography with motility sequences, described in detail below. Inclusion criteria included ability to give informed consent, non-smokers and abstinence from caffeinated and alcoholic drinks on the day of the scan. Exclusion criteria were any known chronic intestinal disease, any long-term medication excluding the oral contraceptive, self-reported gastrointestinal (GI) symptoms or history of GI surgery. Volunteers were recruited prospectively by advertisement and interview. The cohort has previously been reported in a previous study investigating pharmacomodulation of global gut motility.^7^

### Protocol

Volunteers fasted for 4 h prior to ingesting 1 1 of 2% mannitol solution over the 50 min prior to the MRI scan. Subjects ingested the mannitol at regular intervals such that the last of the solution was consumed immediately before entering the scanner room. Subjects lay in the prone position and were scanned using a Philips Achieva® 3T Multi-transmit MRI scanner (Philips Healthcare, Best, Netherlands) using the manufacturer's torso coil (XL-TORSO). Each subject underwent planning sequences followed by a multislice balanced turbo field echo motility sequence (coronal, 2.5 × 2.5 × 5-mm voxel size; field of view, 420 × 420 × 30 mm; flip angle, 20°; echo time = 1.85 ms; repetition time = 3.7 ms dual channel radiofrequency (RF) transmit with adaptive RF shimming), no slice gap with six slices in a volume. Each 3-cm block volume was acquired during a 20-s breath hold (BH) with temporal resolution of 1 volume per second (20 time points acquired per BH). Blocks were acquired sequentially through the abdomen during repeat BHs to cover the whole small bowel volume. The study co-ordinator (AM) selected the volume that best displayed the terminal ileum, and data acquisition was extended to a 1-min period of free breathing (FB).

Each volunteer underwent a second MR examination after a mean gap of 4 weeks (range, 2–7 weeks) using an identical preparation and MRI protocol. The time of day at which time the second scan was undertaken was within 1 h of the first in all cases.

### Region of interest placement

Data sets were anonymized and uploaded to a graphical user interface (GUI) designed in MATLAB^®^ (The MathWorks, Inc., Natick, MA) by the study co-ordinator (AM).

**Figure 1.**
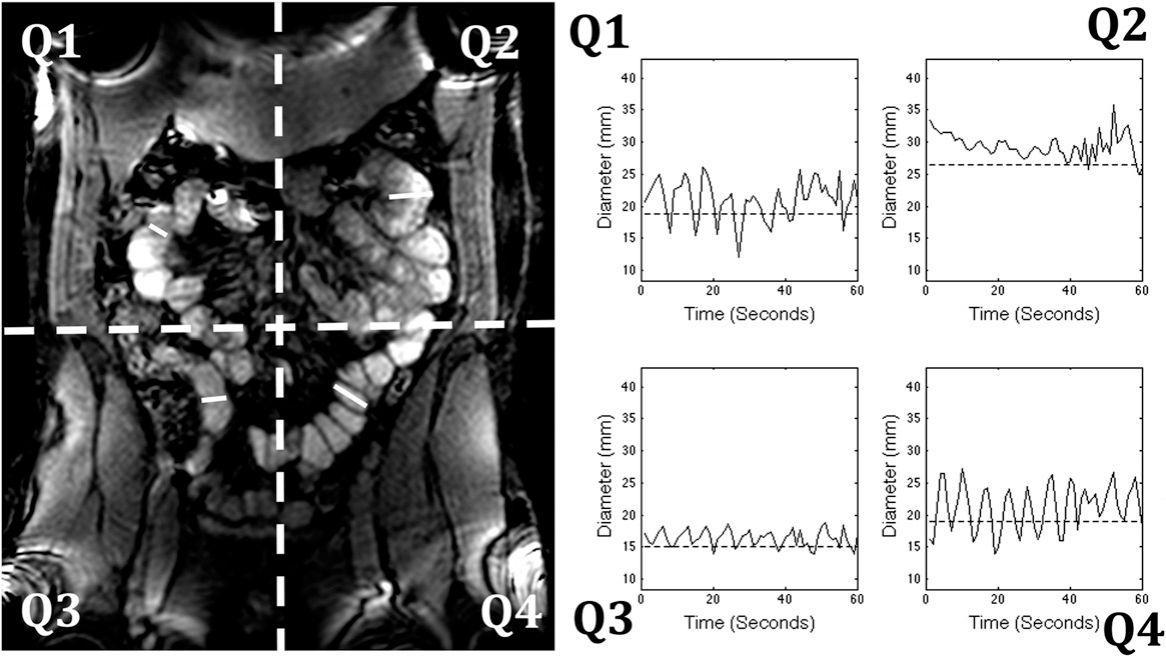
Positions of the four regions of interest in quadrants 1–4 (Q1–Q4) with the terminal ileum in Q3 along with time series plots of line lengths for the respective quadrants.

The coronal cine blocks were divided into four quadrants [upper right (Q1), upper left (Q2), lower right (Q3) and lower left (Q4) by placing two intersecting lines located at the midpoint in the *x* and *y* directions, respectively; Figure 1]. A radiologist with 5 years' experience of MR enterography (AP) not only reviewed all the cine blocks encompassing the small bowel volume and chose the single best block that included the terminal ileum in Q3. In the three other quadrants a loop was selected that could be followed in its entirety throughout the 20-s time series (*i.e.* the loop was well distended and remained in plane on at least one of six slices within the block). For each subject, the observer then placed a linear region of interest (ROI) across the bowel lumen, from bowel wall to bowel wall in a well-distended loop in each of the four quadrants on the most appropriate slice in the block (Figure 1). The Q3 ROI was specifically placed within the last 5 cm of the terminal ileum (Figure 1). The ROI was reproduced by the software across the 20-s time series. The observer then manually adjusted the length and the exact position of each copied ROI where necessary to ensure that it closely followed the lumen of the bowel loop over the 20 s of data acquisition. The process was repeated for the FB data with one ROI in each quadrant as described one ROI previously.

**Figure 2.**
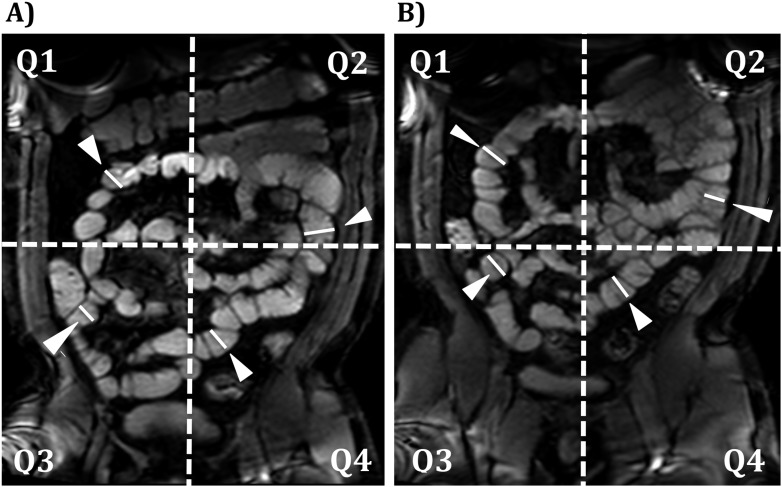
Repeat scans across two time points (a, b) with regions of interest (ROIs) in each quadrant indicated by white arrowheads. Anatomical markers including the ileocaecal valve, duodenojejunal flexure etc. were used to aid ROI duplication. Q1–Q4, quadrants 1–4.

The whole process was repeated for the second scan for each volunteer. The position of each of the four ROIs was replicated as accurately as possible by the observer using knowledge of the location in the *z*-axis of the first block together with visible anatomical landmarks (*e.g.* ileocaecal valve, duodenojejunal flexure etc.) (Figure 2).

### Motility analysis

#### Contractions per minute

Using the GUI, the length of the ROI was plotted against time to create a motility curve. The mean ROI length was calculated, and 10% mean length lines were indicated on the motility curve and small bowel contractions manually counted by the study co-ordinator. A small bowel contraction was recorded where there was a decrease in luminal diameter >10% of the mean small bowel diameter for the given time series.^17^ The contraction rate was expressed in CPM (scaled appropriately for the 20-s BH data). An example time series plot for each quadrant is provided in Figure 1. Contractions were rounded to the nearest integer.

#### Registration-derived motility score

The registration algorithm described by Odille et al^8^ was used as a second method to evaluate motility in both BH and FB data. In summary, each time point in the dynamic series is registered to an automatically selected target frame using an optic-flow technique modified to include contrast changes. Using a multiresolution approach and starting from an initial estimate of zero displacement with no intensity change, the algorithm progresses from coarse to fine scales depicting motion (*e.g.* bowel wall movement) as a deformation between the registration target and a given time point. The deformation fields for each time point was quantified by taking the fractional change in area of a given pixel described by its Jacobian determinant. The SD of this change was taken through time to produce a parametric motility “map” with the pixel values averaged under the manually drawn linear ROI to provide a surrogate for motility. The same technique has previously been used for global analysis by extending a polygonal ROI to cover the whole small bowel instead. The motility score (Jacobian SD) has dimensionless numerical units represented here with the suffix AU (arbitrary unit).

### Statistical analysis

All data were assessed for normality using Shapiro–Wilk testing (alpha *p* < 0.5). No power calculation was performed for this study owing to the absence of available prior data in healthy individuals and the largely descriptive nature of this study.

Correlation between the two motility metrics was assessed using Pearson's correlation in BH and FB data.

The level of agreement between the two motility metrics in each of the four quadrants within a single scan was assessed using intraclass correlation (ICC).

For assessment of intrasubject repeatability across the two scan times, the CPM and Jacobian SD for all four ROIs were assessed with Bland–Altman limits of agreement (BA LoA) adjusted for multiple observations per individual.^18^ This was performed for the BH data and then repeated for the FB data.

All data collection and statistical analysis was performed using MATLAB.

## RESULTS

All subjects achieved adequate distension of the small bowel to allow ROI placement in each of the four quadrants for both scans.

### Correlation between motility metrics

**Figure 3.**
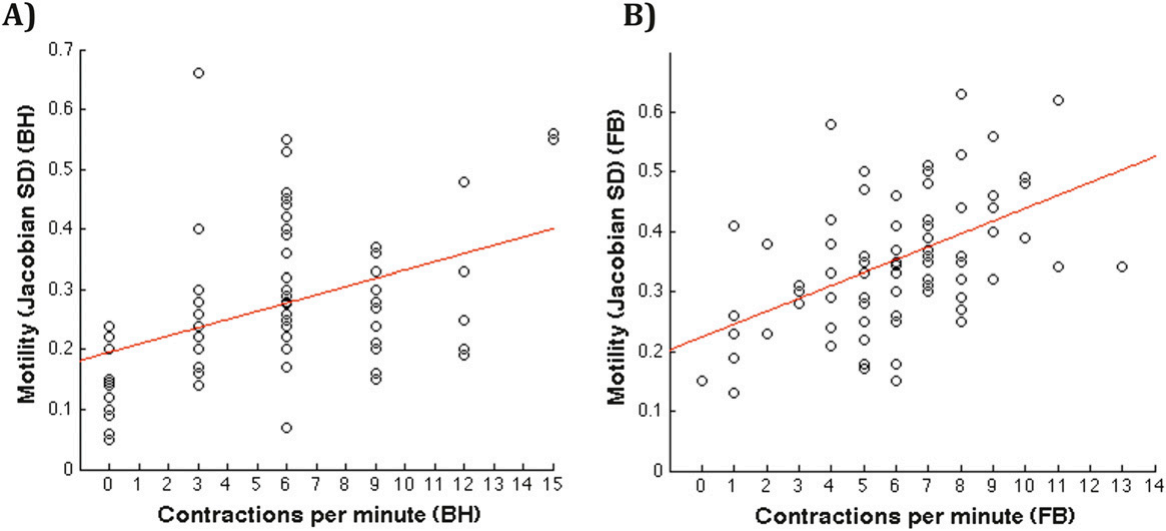
Correlation between contraction rate and standard deviation (SD). Jacobian metric in breath hold (BH) (a) and free breathing (FB) (b).

CPM and the Jacobian SD motility metric showed moderate positive correlation in both BH (Pearson *R* = 0.42; *p* < 0.001; Figure 3a) and during FB (Pearson *R* = 0.58; *p* < 0.001; Figure 3b).

### Within-scan subject variation between different bowel segments

#### Breath-hold results

**Figure 4.**
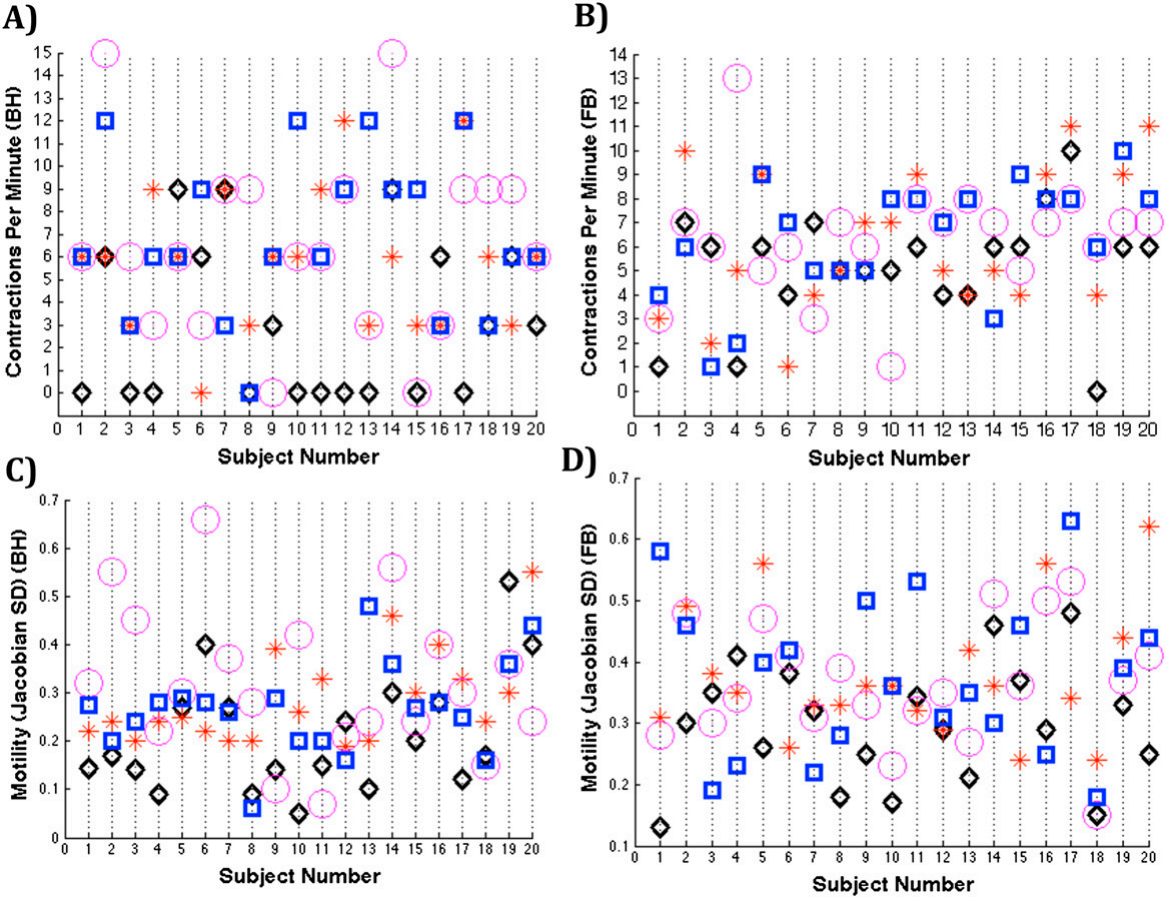
Raw data plots for each subject where “circles” represent quadrant 1 (Q1); “stars”, Q2; “diamonds”, Q3 (terminal ileum); and “squares”, Q4. Contraction rates in breath hold (BH) and free breathing (FB) are shown in (a) and (b) with respective plots for Jacobian standard deviation (SD) in (c) and (d).

The CPM and Jacobian SD mean, range and SD for each of the four quadrants is summarized in Table 1. Assessment with ICC in BH data demonstrated a *R*-coefficient of 0.06; *p* = 0.1. Individual data points for each subject are presented in Figure 4a. Similar assessment of the Jacobian SD motility metric demonstrated a significant but weak ICC *R*-coefficient = 0.2; *p* = 0.027 (Figure 4c). These data broadly suggest high variation in both contraction rate and motility score between quadrants in the same individual.

**Table 1.** Summary data for intrascan variation across free-breathing (FB) and breath-hold (BH) protocols for contraction rate and Jacobian standard deviation (SD) metrics

Mean motility (CPM/Jacobian SD)	Mean	Range	SD
BH: CPM			
Q1	6.00	0.00–15.00	1.00
Q2	5.00	0.00–12.00	1.00
Q3 (TI)	3.00	0.00–9.00	2.00
Q4	6.00	0.00–12.00	1.00
BH: motility AU (Jacobian SD)			
Q1	0.29	0.09–0.66	0.10
Q2	0.32	0.20–0.53	0.15
Q3 (TI)	0.21	0.05–0.51	0.12
Q4	0.27	0.06–0.47	0.10
FB: CPM			
Q1	6.00	1.00–13.00	3.00
Q2	6.00	1.00–11.00	2.00
Q3 (TI)	6.00	0.00–10.00	3.00
Q4	6.00	1.00–10.00	3.00
FB: motility AU (Jacobian SD)			
Q1	0.38	0.13–0.53	0.10
Q2	0.37	0.24–0.62	0.10
Q3 (TI)	0.29	0.15–0.49	0.10
Q4	0.37	0.18–0.63	0.13

AU, arbitrary unit; CPM, contractions per minute; Q1–4, quadrant 1–4; TI, terminal ileum.

For example, the CPM variation within volunteer 13 was 0 CPM in Q2 and 12 CPM in C4, while the total range across the cohort in CPM was 0–15 CPM. Similarly, for the Jacobian SD metric, Subject 2 had motility values in Q2 of 0.15 AU and 0.55 AU in Q1, while the range across the cohort was 0.05–0.66 AU.

#### Free-breathing results

The mean, range and SD of each quadrant's contraction rate over the 60-s FB period are summarized in Table 1. Individual data points for each subject are presented in Figure 4b,d.

In general, the magnitude of CPM was similar to that acquired during a 20-s BH (Table 1), although the variation within individuals was reduced (Figure 4b,d). Notably, the ICC demonstrated a marginally increased and significant *R*-coefficient of −0.26; *p* = 0.05. The result for the Jacobian motility metric remained similar with a weakly significant *R* coefficient of 0.19; *p* = 0.03. In the BH data, there was high variation between quadrants in individual volunteers. For example, Subject 1 had Q3 and Q4 motility of 0.13 and 0.58 AU, while the maximum and minimum motility across the cohort ranged between 0.13 and 0.62 AU.

### Within-subject repeatability between different time points

#### Breath-hold results

**Figure 5.**
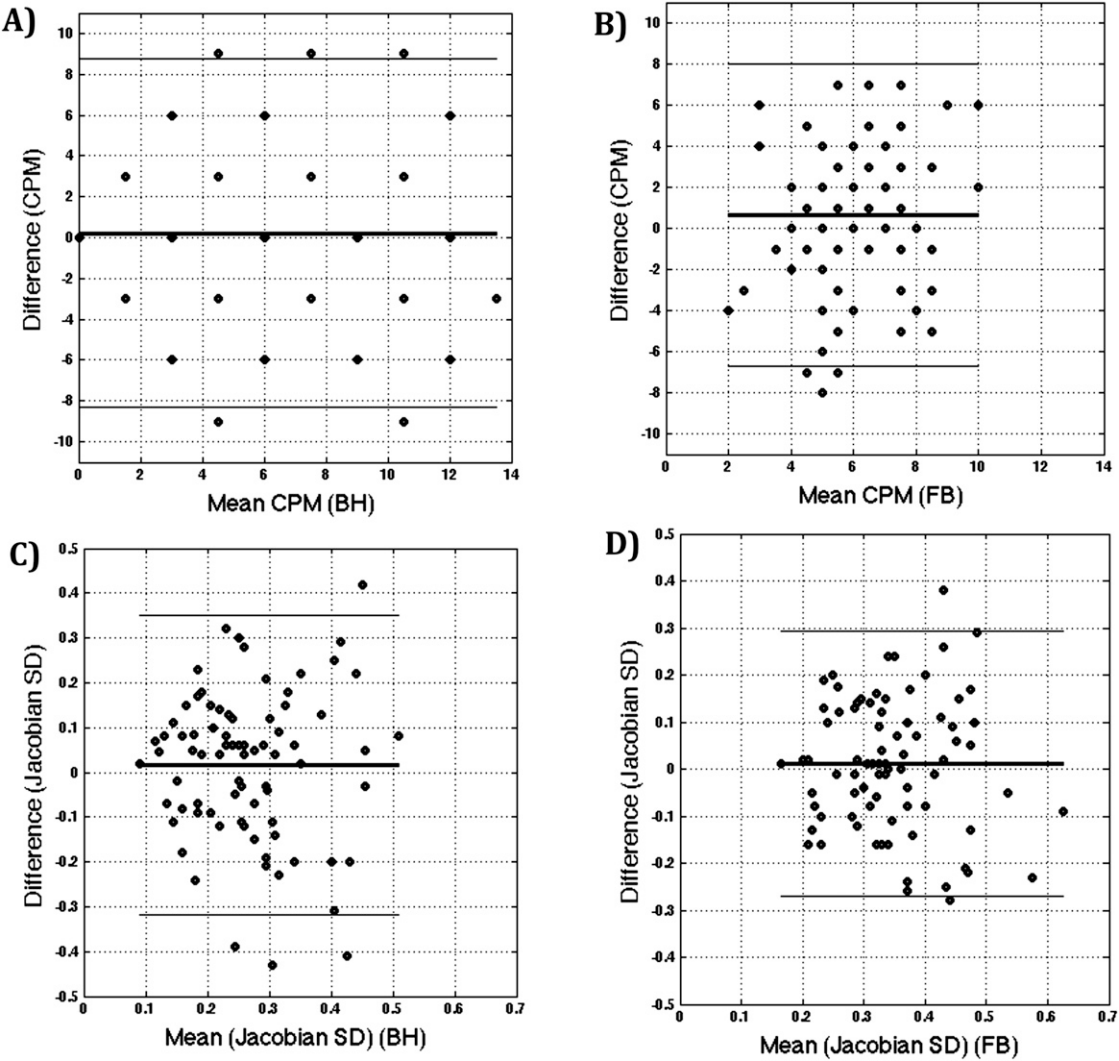
Bland–Altman limits of agreement adjusted for repeat observations per individual, for contraction rate in breath hold (BH) and free breathing (FB) (a, b). Respective motility scores with Jacobian standard deviation (SD) metric (c, d). CPM, contractions per minute.

Across all ROIs in all subjects, the mean CPM at time point 1 was 6 contractions (range, 0–15) and the mean CPM at time point 2 was 6 contractions (range, 0–13). The BA LoA for repeat measures demonstrated a mean difference of 0 contractions with a LoA of ±8 contractions (Figure 5a), suggesting relatively poor repeatability of CPM across time for matched segments.

Using the Jacobian SD metric, the mean motility score at time point 1 was 0.28 AU (range, 0.05–0.66) and the mean motility at time point 2 was 0.26 AU contractions (range, 0.07–0.63). The BA LoA for repeat measures demonstrated an mean difference of 0.01 contractions with an LoA of ±0.32 contractions (Figure 5c), suggesting again a large source of intrasubject variability.

#### Free-breathing results

The mean contraction number at time point 1 was 6 contractions (range, 0–13) and mean contraction at time point 2 was 6 contractions (range, 0–11). The BA LoA for repeat measures demonstrated a mean difference of 1 contraction with a LoA of ±6 contractions (Figure 5b), suggesting relatively poor repeatability of CPM across time.

Using the Jacobian SD metric, the mean motility score at time point 1 was 0.35 AU (range, 0.05–0.63 AU) and the mean CPM at time point 2 was 0.34 AU contractions (range, 0.13–0.69 AU). The BA LoA for repeat measures demonstrated a mean difference of 0.01 contractions with an LoA of ±0.32 contractions (Figure 5d), suggesting a large source of intrasubject variability.

## DISCUSSION

This prospective study aimed to examine variation of segmental SBM in a cohort of 20 healthy volunteers. In summary, large variation in segmental motility within the same individual was demonstrated both at different small bowel locations and at the same location at different times. This raises important methodological limitations when performing segmental analysis of bowel motility, regardless of the metric used.

We employed both BH and FB protocols to replicate the majority of study protocols described in the literature and to test the influence of breathing protocols on the motility scores reported. Intuitively, it might be presumed that FB data are better suited for CPM analysis, as the longer period of data collection makes it more robust to potential transient su rges in contractile activity.^19,20^ Indeed, we saw evidence for this with longer acquisition times where there was decreased intrasubject variance and an increased ICC. Nevertheless, even with 60 s of FB, there remained large variation in the segmental motility across small bowel quadrants and between different scan times.

With FB appearing superior for the CPM metric, BH studies might appear redundant. However, there are several potential advantages. First, a 20-s BH is rapid and, if the aim is to sample the whole small bowel volume (requiring multiple acquisitions), a smaller scan time is preferable, particularly if translated into clinical practice. Again from a practical perspective, the BH protocol reduces the data volume generated and allows more feasible analysis with manual quantitation or post processing. Furthermore, the anteroposterior displacement caused by the respiratory cycle can “remove” sampled small bowel loops for periods within the time series. BHs reduce/eliminate displacement caused by respiration, which ensures any bowel loop can be sampled and not just those well seen throughout the time period, which may reduce sampling bias. Many MRI motility metrics have been developed to work over these shorter time periods including the Jacobian SD measure used here, where the SD rather than the sum of the small bowel movement is recorded. Analogous metrics using bowel calibre data have also been described (*e.g.* contraction amplitude) where only a single, well-formed contraction is sampled independent of time assuming the scan is long enough to fully resolve one contraction.^4,5,14^ The present study saw a negligible reduction in variance for the Jacobian SD metric between BH and FB protocols. For longer time periods, the Jacobian metric could be standardized, as the contraction rate has been, to a period of 1 min; for example, this has not been performed here as we wanted to use existing, validated metrics.

By placing a ROI in each quadrant, we could examine the range of motility within subjects at a single point in time and found that there was in fact large variation within the different segments of the bowel. Further examination of the data graphically and visually confirmed the apparent heterogeneity of contractions through the bowel, raising an important point for further consideration. In several recent studies, subjective selection of bowel loop based on good distension has been performed and assumed to be representative of global motility, for example, higher motility in this loop being attributed to a non-diseased state.^4,14,15^ Across both BH and FB, we saw in many of the subjects both relatively “hypomotile” and “hypermotile” segments based on both their CPM and Jacobian SD scores. This within-subject heterogeneity presents a serious challenge to the persevering notions of hyper-/hypomotile bowel derived from an analysis of limited small bowel loops. The fact that both metrics provided comparable ICC scores and spread of data values implies this reflects genuine physiological variability and is not particular to any specific metric. The results presented here are principally descriptive but may serve a role in determining study power in future investigations examining segmental dysmotility in health and disease.

The final result presented in this study describes poor intrasubject repeatability of the CPM and Jacobian SD metrics at time points on average 4 weeks apart. This held true for both BH and FB data sets. Indeed, intrasubject variation over time appeared similar in magnitude to between subject variation for several participants in the cohort. The study cohort was standardized for a number of factors that might affect SBM with particular attention paid to preparation with oral contrast agent, caffeine and nicotine intake, time of day and ingestion of medication. One of the principal influencing factors for the variation might be variation in ROI placement between scans. Although care was taken to duplicate precisely the ROI position across the quadrants, it was in fact difficult in the absence of *in situ* markers, to be certain of the exact location of the previous ROI. In this respect, we ensured one ROI for each scan was placed within 5 cm of the terminal ileum where a high degree of certainty could be achieved with respect to the repositioning of the ROI. Despite this, at the terminal ileum, we again saw high variation between scans both during BH and FB across the two metrics.

With respect to limitations, 60 s of FB might still be argued to be too short a time to evaluate motility with respect to CPM and extended FB studies should be conducted to evaluate the impact on repeatability and reproducibility of analysis. Another limitation lies in the assessment of only two metrics in this study with still no clear consensus for the usage of certain metrics, including mean diameter and contraction amplitude, established. Additionally, the cohort described in this study is relatively small and homogeneous in terms of age. A larger sample size would assist in the better characterization of motility in healthy subjects using MRI.

Whether or not segmental metrics are useful for the investigation of SBM is unclear. Undoubtedly, where ROI placement can be guided by the existence of pathology (such as a stricture), segmental methods of analysis are valid and, as scanner hardware and software improves, this form of analysis will likely become increasingly valuable for the investigation of diseases affecting specific bowel segments. Indeed, several studies have already shown an inverse relationship between segmental bowel motility and inflammatory activity in terminal ileal Crohn's disease.^2,3^ However, owing to the apparent heterogeneity within normal bowel, great caution must be placed on inferences based on analysing subjectively placed segmental ROIs. Previously, Menys et al^7^ has demonstrated good repeatability using a technique that captures global motility of the whole small bowel volume using the metric based on Jacobian SD. In this work, the reproducibility of global motility between the two scans was superior to the segmental data presented in the present study with BA LoA at ±0.044 across a cohort range of 0.12 AU, thereby demonstrating smaller variability within than between subjects. Other studies have also applied techniques that sample volume rather than segmental data. For example, van der Paardt^10^ applied a tagged sequence to acquire motility from small bowel volume without the need for segmental ROI placement.^16^ Combined with the data of the present study, this suggests global analysis of whole small bowel volumes will be more robust than segmental sampling, particularly for investigating enteric motility disorders.

## CONCLUSION

MRI-quantified normal segmental SBM demonstrates wide variation across different locations within the small bowel and in the same location at different time points. This may limit application of segmental analysis for the investigation of global dysmotility.

## FUNDING

This work was undertaken at the University College London Hospital/University College London who received a proportion of funding from the Department of Health's National Institute for Health Research Biomedical Research Centre's funding scheme grant. SA Taylor is an National Institute of Health Research senior investigator.
